# 1q25.2-q31.3 Deletion in a female with mental retardation, clinodactyly, minor facial anomalies but no growth retardation

**DOI:** 10.1186/1755-8166-6-30

**Published:** 2013-08-06

**Authors:** Ping Hu, Yan Wang, Lu-lu Meng, Ling Qin, Ding-yuan Ma, Long Yi, Zheng-feng Xu

**Affiliations:** 1 State key Laboratory of Reproductive Medicine, Department of Prenatal Diagnosis, Nanjing Maternity and Child Health Care Hospital Affiliated to Nanjing Medical University, 123 Tianfei Street, Nanjing 210029, China; 2 Department of Pathology, Nanjing University Medical School, Nanjing 210093, PR China

**Keywords:** Interstitial 1q deletion, SNP-array, Mental retardation, Growth retardation

## Abstract

The reports of 1q25-32 deletion cases are rare. We reported here an 11-year-old Chinese Han female with an interstitial 1q25 deletion displaying mental retardation, clinodactyly of the 5th finger and minor facial anomalies. Notably, the patient did not present growth retardation which is quite common in patients with 1q25-32 deletion encompassing LHX4. The heterozygous deletion in this patient was characterized as 46,XX,del(1)(q25.2-q31.3) with a length of 20.5 Mb according to SNP-array test results. STRP (Short Tandem Repeat Polymorphism) analysis of the family trio indicated the genomic abnormality was *de novo* with paternal origin. After a genotype-phenotype analysis, we proposed here the loss of a 3.1 Mb critical region including 24 genes within 1q25.2 (chr1:174.5-177.6 Mb, build 36) may account for the mental retardation in patients with 1q25-32 deletion.

## Background

The reports of chromosome 1q deletion have been relatively rare. Based on the deletion region, interstitial 1q deletions are conventionally classified into three categories: proximal (1q21-22q25), intermediate (1q24-25q32), and distal (1q42-43qter) deletion [1]. Up to date, about 25 intermediate 1q25-q32 deletion cases have been documented, of which, only 15 cases [2-12] were well characterized using high-resolution molecular methods to map the accurate deletion sizes. In the current study, a heterozygous 1q25.2-q31.3 deletion in a female of 11-year-old presenting mental retardation but no apparent growth delay was characterized by SNP-array.

### Clinical report

The proband, an 11-year-old Chinese Han girl, is the first child born to healthy and non-consanguineous 25-year-old mother and 24-year-old father. She was delivered at 39 weeks of gestation by cesarean due to fetal distress. The pregnancy was uneventful with normal ultrasound and serum examinations reports throughout the pregnancy. The patient was born with a weight of 2,730 g (−1.3SD), a length of 48 cm (−1.1SD), a head circumference (OFC) of 35 cm (+0.9SD), and Apgar score of 10 at 1 minute and 10 at 5 minutes. Birth weights of the father and mother were 2,680 g (−1.55SD) and 3,000 g (−0.5SD) while birth lengths and OFC were unavailable. The mother was a clerk and the father was a businessman, both with high school diploma as a terminal degree. There was no intellectual disability history in the family.

She rolled over at 4 months, sat without support at 7 months, crawled at 9 months. Psychomotor retardation was recognized due to her delayed walking when she was 18 months old. She spoke her first words at age of 4.5 years after a special training. At the age of 9, she was evaluated with a full scale IQ of 49, performance IQ of 53 and verbal IQ of 54 by Wechsler Intelligence Scale for Children.

**Figure 1:**
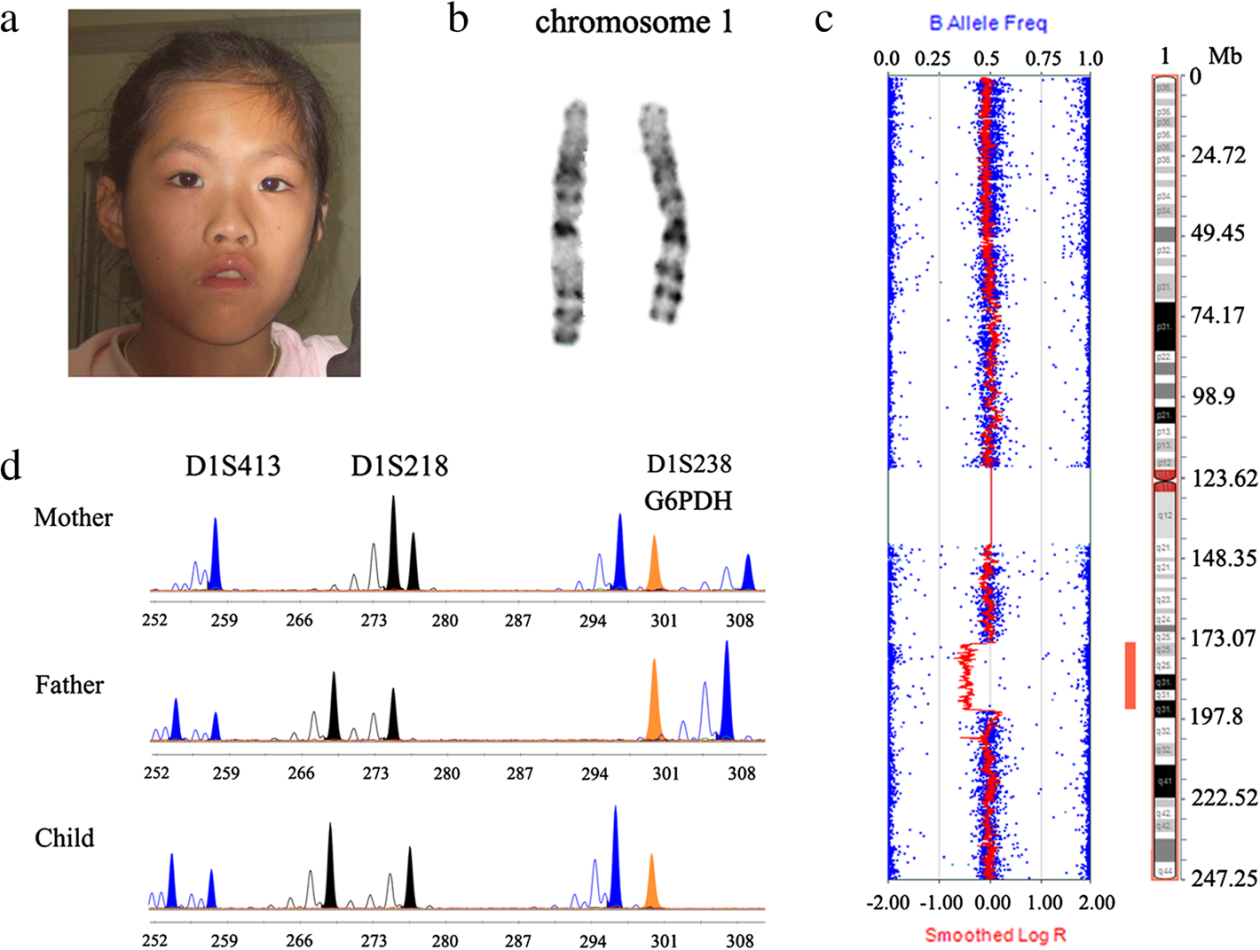
**Identification of a 1q25.2-31.3 deletion in the proband. a:** Facial image of the patient at the age of 11. She showed prominent forehead, upslanting palpebral fissures, short philtrum, everted upper lip, hypertelorism, broad and depressed nasal bridge, arched eyebrows. **b:** Two homologous chromosomes 1 of the patient. **c:** SNP-array analysis revealed a 1q deletion spanning from 174,592,050-195,122,910 with a length of 20.5 Mb. **d:** Competitive fluorescent multiplex STRP assay demonstrated the deletion was of paternal origin. Three high informative STR sites D1S238 (chr1: 186412815–186413111), D1S218 (chr1: 172769757–172770032) and D1S413 (chr1:196887255–196887453) and G6PDH were analyzed, results of D1S238 indicated the loss of the paternal allele in the female.

The proband was referred to our genetic diagnosis center when she was 11 years old. Her weight was 23 kg (−1.74SD), height 134 cm (−1.67SD), and OFC 52 cm (−0.6SD), without apparent growth retardation. The father was 68 kg (+1.72SD) in weight and 169 cm (+0.12SD) in height at age of 35, and the mother was 55 kg (+0.76SD) and 159 cm (+0.34SD) at age of 36. The anticipated mid-parental height of the patient was 157.5 cm (+0.07SD), in accordance with her current height (−1.67SD). Her hands and feet sizes were in an ordinary range (25th-75th percentile), with a palmar length of 8.5 cm, middle finger length 6.3 cm and foot length 20.5 cm. Minor anomalies including prominent forehead, upslanting palpebral fissures, short philtrum (philtrum length 1.1 cm, < 3th percentile), everted upper lip, low posterior hairline, hypertelorism (inner canthal distance 3.7 cm, > 97th percentile), broad and depressed nasal bridge, arched eyebrows (Figure 1a) as well as bilateral clinodactyly of 5th finger were observed. Her palate appeared normal without clefts, and there was no thyromegaly or congenital webbed neck. Neither hearing impairment nor otitis media was found. Ophthalmologic examination results were normal. Heart murmurs were not detected via cardiac auscultation. Radiographic examination confirmed bilateral clinodactyly of 5th finger, and no abnormalities were found on phalanges or metacarpals. Her bone age was consistent with the chronological age. The spine didn´t show any distinctive radiographic results. Electroencephalograph showed an abnormal brain-wave activity, mainly focusing on wave θ. Ultrasonography results indicated her uterus and ovaries were normal in size and other internal organs including liver, spleen, pancreas and kidneys appeared normal. Head magnetic resonance imaging and two-dimensional echocardiography did not reveal any anomaly. Serum GH, TSH, fT3, and fT4 levels were all in normal range. She was unable to dress herself. She spoke with a lisp and had trouble in communication. This work was approved by the Medicine Ethics Committee of Nanjing Maternity and Child Health Care Hospital and the proband's legal guardian signed informed consent.

## Results

### Cytogenetic analysis

GTG-banding on the patient’s peripheral blood lymphocytes revealed an interstitial 1q deletion, the karyotype was designated as 46,XX,del(1)(q25-q31) (Figure 1b). The karyotypes of the parents were normal.

### SNP-array and STRP analysis

A 20.5 Mb deletion (chr1:174,592,050-195,122,910, build 36) within 1q25.2-q31.3 was detected from the patient’s sample (Figure 1c). SNP-array analysis on the parents did not reveal any pathological CNV (copy number variation), indicating the deletion was de novo. SNP genotyping analysis of the family trio was performed to explore the parental origin of the deleted 1q25.2q31.3 fragment. Additional file 1: Table S1 listed representative 29 sites selected from thousands of homozygote SNP sites within and around the breakpoints, presenting different genotypes between the parents. Based on the data from these homozygote SNP sites, we concluded the lost allele was of paternal origin. Microsatellite analysis using D1S413, D1S218 and D1S238 also confirmed the deletion was of paternal origin (Figure 1d). Since the deletion was *de novo*, the recurrence possibility of the deletion in the next pregnancy of the couple is theoretically low. In accordance with the prediction, the prenatal genetic diagnosis on the amniotic fluid cells using SNP-array indicated the following baby of the parents did not carry the 1q25.2q31.3 deletion as well as any other pathological genomic imbalance.

## Discussion

In this study, we have identified a *de novo* paternal interstitial deletion of chromosomal segment 1q25.2-q31.3 with a length of 20.5 Mb in an 11-year-old female presenting mental retardation but no apparent growth delay by GTG banding, SNP-array and STRP analysis.

**Figure 2:**
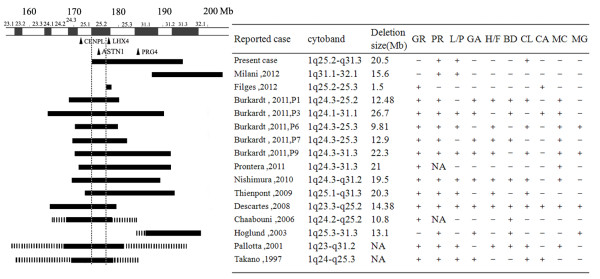
**Genotypes and phenotypes of the 16 previously identified cases of 1q25-32deletions.** The locations of deletions are listed in the left. Deletions confirmed by molecular methods are illustrated with solid horizontal lines and unconfirmed deletions with dotted line. Phenotypes of each deletion are listed in right box. GR, growth retardation; PR, psychomotor retardation; L/P, lip/palate anomalies; GA, genital abnormalities; H/F, small hands/feet; BD, brachydactyly; CL, fifth finger clinodactyly; CA, cardiac anomalies; MC, microcephaly; MG, micrognathia; +, positive; -, negative; NA, no available; P, patient.

The most common features of intermediate 1q deletion patients are pre- and/or postnatal growth retardation, psychomotor retardation, lip and palate anomalies, genital abnormalities, small hands and feet, brachydactyly, clinodactyly of the fifth finger, cardiac anomalies, microcephaly, micrognathia (Figure 2). The sizes and locations of two deletion cases reported by Burkardt (patient 9) [10] and Thienpont [7] are quite similar to ours. Burkardt [10] reported an 8-year-old boy with a 22.3 Mb 1q24.3-31.3 deletion who manifested growth retardation, psychomotor retardation, microcephaly, small ears, bilateral cleft lip and palate, micrognathia, loose nuchal skin, cryptorchidism, inguinal hernia, short limbs, small hands and feet, fifth finger clinodactyly and hypotonia. Thienpont [7] identified a 20.3 Mb 1q25.1-31.3 deletion in an 11-year-old boy who manifested growth retardation, psychomotor retardation, recurrent otitis media, narrow high-arched and triangular shaped palate, small hands and feet, fifth finger clinodactyly, abducted thumbs, persistent finger pads, 2–3 partial syndactyly of right foot, severe myopia and mild strabismus. Unlike these two cases, the manifestations of our patient are remarkably milder. She presented only mental retardation, clinodactyly of the 5th finger and minor facial anomalies, but no obvious growth retardation.

Except for two cases reported by Hoglund and Milani [4,12], severe pre- and/or postnatal growth retardation was found in almost all of the patients with 1q25-q32 deletion. LHX4 (LIM homeobox gene 4) and CENPL (centromeric protein L) were suggested to be the primary candidate genes for growth retardation in the 1q25-q32 region. Initially, several studies indicated that growth retardation was likely attributed to the low serum GH levels resulting from heterozygous deletion of LHX4 in patients [6-8]. Heterozygous mutations of LHX4 can lead to abnormalities of pituitary and cerebellum, the deficiency of growth hormone, and then growth retardation [13]. Two individuals of 1q25q32 deletion with LHX4 loss but no CENPL loss displayed significant growth retardation [7,11]. However, there were several discordant reports regarding LHX4 deletion and growth retardation [10,11]. Filges reported a female neonate with a maternally inherited 1q25.2q25.3 deletion affecting LHX4 but not CENPL had GH deficiency, but her mother was phenotypically normal [11]. Intriguingly, a recent report suggested CENPL deletion was responsible for growth retardation [10], because nearly all of the documented cases with interstitial 1q deletion harboring CENPL gene presented growth retardation. Burkardt [10] reported a cohort of nine individuals with 1q24q25 deletions manifesting growth retardation. Four of them did not show a loss of LHX4, while all of the nine deletions showed a loss of CENPL. CENPL, encoding centromeric protein L, has been proved to play an important role in assembling a functional kinetochore and mitosis process [14], but the mechanism underlying CENPL deletion and growth retardation remain elusive. Taken together, CENPL heterozygous deletion exhibited a full penetrance to growth retardation, while LHX4 deletion displayed an incomplete penetrance. Two patients with 1q25q32 deletion didn’t exhibit growth retardation [4,12], may due to the fact that their deleted regions encompassed neither LHX4 nor CENPL. Likewise, the normal growth in our patient was probably due to the fact that CENPL gene did not locate in the deleted region.

Mental retardation is the most frequent manifestation of 1q25-32 deletion patients, but the causative genes have not been identified. Based on the genotype-phenotype analysis, we proposed a critical region at 1q25.2 (chr1:174.5-177.6 Mb, build 36) with a length of 3.1 Mb may account for mental retardation. Eleven postnatal cases with 1q25-32 deletion containing the proposed critical region displayed apparent mental retardation (Figure 2), while two of the remaining three 1q25-32 deletions outside of the critical region exhibited mild mental retardation [4,12] and another case of neonate is absent from mental retardation [11]. Twenty-four genes located in the critical region, including 7 OMIM genes (ASTN1, RASAL2, ANGPTL1, FAM20B, TOR3A, ABL2, SOAT1). ASTN1 (Astrotactin 1), the only one reported to be involved in the brain development, is essential for glial-guided migration in the brain cortex [15]. ASTN1 mutations were probably responsible for a spectrum of cortical malformations including lissencephaly, pachygyria, abnormally thick cortex, enlarged lateral ventricles, hypoplasia of the corpus callosum/cerebellum in human [16]. Mice with null ASTN1 showed smaller sizes of cerebella, poorer balance and coordination, reduction in migration rates [17]. Of the eleven postnatal deletion cases harboring ASTN1 [2,3,6-8,10], six displayed brain structure malformations [2,3,7,10], four were normal by MRI analysis [8,10], and the remaining one is unknown. We concluded that heterozygous ASTN1 deletion could be an important cause of the mental retardation in 1q25-32 deletion patients.

Clinodactyly 5th finger was a common clinical feature of 1q25q32 deletion cases. PRG4 (proteoglycan 4) encodes a secreted glycoprotein Lubricin, which exists in synovial fluid and the surfaces of articular cartilage, protecting the cartilage surfaces [18]. Homozygous PRG4 mutations in human lead to CACP (autosomal recessive camptodactyly-arthropathy-coxa vara-pericarditis syndrome) [19], while mice with null PRG4 alleles (PRG4^−/−^) exhibited morphologic joints changes such as joints swelling and flexion deformity of the hind paws [18]. Two of four previously reported postnatal patients with PRG4 deletion manifested joint abnormalities [8,10]. Though PRG4 haploinsufficiency showed a strong association with skeletal abnormalities in 1q25-32 deletion patients, there was no direct evidence linking the PRG4 deletion and clinodactyly 5th finger to date.

In this study, we determined the paternal origin of the deletion by SNP genotypes comparison and STRP tests. As far as we know, 7 intermediate 1q deletions (including ours) were tested by microsatellite analysis to investigate the origin of the genomic deletion. Only one deletion [3] was derived from mother, the remaining six [4-6,8,12] were all of paternal origins. However, distinctive features between the 1q25-32 deletion patients who have different parental origin were not observed. This phenomenon coincides with previous reports on other symptoms resulted from loss of heterozygosity in this region, which didn’t find imprinted genes either [20,21]. To our best knowledge, no imprinted gene has been found in the region of 1q25-32 yet. These findings indicate a possible irrelevance between the clinical manifestations and parental origin of 1q25-32 deletion.

## Conclusion

In this study, using SNP-array, we characterized a 1q25-32 deletion case with mental retardation but no significant growth delay. SNP-array and microsatellite analysis revealed the deletion was derived from the father. Our study provided another essential case for further genotype-phenotype correlation studies. However, the mechanisms underlying mental retardation resulting from 1q25-32 deletion are far from clear, more related cases as well as functional genome studies in the future would be helpful to decipher the problem.

## Materials and methods

### Cytogenetics

GTG-banding chromosome analyses at 500 band resolution were performed on the peripheral blood lymphocytes from the patient and her parents according to the standard techniques.

### SNP-array

Human cyto12 SNP-array (Illumina, USA) comprising around 300,000 SNPs was applied for whole genome scan on the patient and parents. SNP-array experiments were carried out as previously described [22], molecular karyotype and SNP genotype analysis was performed by KaryoStudio V 1.3.11(Illumina) and GenomeStudio V2011.1(Illumina) respectively.

### STRP

To confirm the parental origin of the deleted 1q25.2q31.3 fragment, STRP analysis was performed following a previously described protocol [23]. Three STRP markers with high heterozygosity (D1S413, D1S218 and D1S238) in the 1q25q32 region were applied.

### Consent

Written informed consent to participate in this study and to publish photographs was obtained from a parent of the patient.

## Competing interests

The authors declare they have no competing interests.

## Authors’ contributions

PH and YW interpreted the data of SNP-array and drafted the paper. LLM and LQ performed SNP-array. DYM was responsible for the conventional and molecular cytogenetic analysis. LY conducted and interpreted STRP. ZFX designed the study and gave the final approval of the manuscript. All of the authors read and approved the final manuscript.

## Supplementary Material

Additional file 1: Table S1 B allele SNP genotypes comparison analysis. Click here for file
