# Supplementary material for: 1q25.2-q31.3 Deletion in a female with mental retardation, clinodactyly, minor facial anomalies but no growth retardation
Source: Mol Cytogenet. 2013 Aug 6;6:30. doi: 10.1186/1755-8166-6-30 (PMC3766032; doi:10.1186/1755-8166-6-30)
Supplement: Additional file 1: Table S1 — B allele SNP genotypes comparison analysis. [file 1755-8166-6-30-S1.doc]

Table 1 B allele SNP genotypes comparison analysis

| **SNP** | **Chr**  **Location** | **Cyto**  **Band** | **deletion region** | **Genotype** | | |
| --- | --- | --- | --- | --- | --- | --- |
|  |  |  |  | child | mother | father |
| **rs6665614** | 171721230 | 1q24.3 | out | AB | AA | BB |
| **rs2207061** | 173055352 | 1q25.1 | out | AB | AA | BB |
| **rs16845308** | 173009832 | 1q25.1 | out | AB | BB | AA |
| **rs6670617** | 174246712 | 1q25.1 | out | AB | BB | AA |
| **rs10912773** | 174343705 | 1q25.1 | out | AB | BB | AA |
| **rs6665385** | 176114487 | 1q25.2 | in | A | AA | BB |
| **rs520661** | 176129276 | 1q25.2 | in | A | AA | BB |
| **rs596859** | 176167947 | 1q25.2 | in | B | BB | AA |
| **rs2039230** | 176174791 | 1q25.2 | in | A | AA | BB |
| **rs704834** | 176189141 | 1q25.2 | in | B | BB | AA |
| **rs10798450** | 176268044 | 1q25.2 | in | B | BB | AA |
| **rs4651025** | 179371973 | 1q25.2 | in | B | BB | AA |
| **rs4133136** | 179376896 | 1q25.2 | in | A | AA | BB |
| **rs1410594** | 179596179 | 1q25.2 | in | A | AA | BB |
| **rs621071** | 182682811 | 1q25.3 | in | B | BB | AA |
| **rs2482806** | 183684087 | 1q25.3 | in | B | BB | AA |
| **rs7556537** | 185833565 | 1q25.3 | in | A | AA | BB |
| **rs12726519** | 186898065 | 1q25.3 | in | B | BB | AA |
| **rs6697145** | 186910591 | 1q25.3 | in | B | BB | AA |
| **rs10920995** | 188946679 | 1q31.3 | in | A | AA | BB |
| **rs12403168** | 191571287 | 1q31.2 | in | B | BB | AA |
| **rs16835319** | 193334309 | 1q31.2 | in | A | AA | BB |
| **rs877913** | 193408053 | 1q31.2 | in | B | BB | AA |
| **rs10429908** | 194032486 | 1q31.3 | in | A | AA | BB |
| **rs488612** | 194041058 | 1q31.3 | in | A | AA | BB |
| **rs9659527** | 196418527 | 1q31.3 | out | AB | BB | AA |
| **rs10922071** | 196433870 | 1q31.3 | out | AB | BB | AA |
| **rs4462080** | 196567597 | 1q31.3 | out | AB | BB | AA |
| **rs12405238** | 196661613 | 1q31.3 | out | AB | AA | BB |

B allele genotypes of SNPs within and around the deletion from the trio were analyzed. 29 SNPs in a homozygous opposite state in the parents were listed.
